# Noise in Otolaryngology – Head and Neck Surgery operating rooms: a systematic review

**DOI:** 10.1186/s40463-020-00487-6

**Published:** 2021-02-11

**Authors:** Gianluca Sampieri, Amirpouyan Namavarian, Marc Levin, Justine Philteos, Jong Wook Lee, Anni Koskinen, Vincent Lin, John Lee

**Affiliations:** 1 Faculty of Medicine, University of Toronto, Toronto, ON Canada; 2 Department of Otolaryngology Head and Neck Surgery, University of Toronto, Toronto, ON Canada; 3 Department of Otorhinolaryngology, University of Helsinki and Helsinki University Hospital, Helsinki, Finland

**Keywords:** Surgical safety, Operating room communication, Noise in the operating room

## Abstract

**Objective:**

Noise in operating rooms (OR) can have negative effects on both patients and surgical care workers. Noise can also impact surgical performance, team communication, and patient outcomes. Such implications of noise have been studied in orthopedics, neurosurgery, and urology. High noise levels have also been demonstrated in Otolaryngology-Head and Neck Surgery (OHNS) procedures. Despite this, no previous study has amalgamated the data on noise across all OHNS ORs to determine how much noise is present during OHNS surgeries. This study aims to review all the literature on noise associated with OHNS ORs and procedures.

**Methods:**

Ovid Medline, EMBASE Classic, Pubmed, SCOPUS and Cochrane databases were searched following PRISMA guidelines. Data was collected on noise measurement location and surgery type. Descriptive results and statistical analysis were completed using Stata.

**Results:**

This search identified 2914 articles. Final inclusion consisted of 22 studies. The majority of articles analyzed noise level exposures during mastoid surgery (18/22, 82%). The maximum noise level across all OHNS ORs and OHNS cadaver studies were 95.5 a-weighted decibels (dBA) and 106.6 c-weighted decibels (dBC), respectively (*P* = 0.2068). The mean noise level across all studies was significantly higher in OHNS cadaver labs (96.9 dBA) compared to OHNS ORs (70.1 dBA) (*P* = 0.0038). When analyzed together, the mean noise levels were 84.9 dBA.

**Conclusions:**

This systematic review demonstrates that noise exposure in OHNS surgery exceeds safety thresholds. Further research is needed to understand how noise may affect team communication, surgical performance and patient outcomes in OHNS ORs.

**Graphical abstract:**

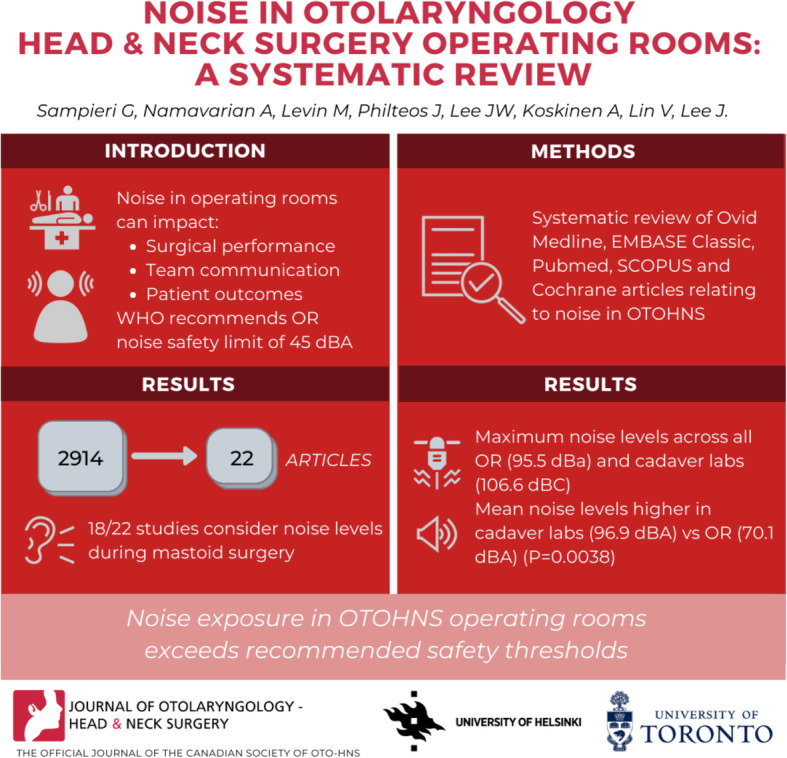

**Supplementary Information:**

The online version contains supplementary material available at 10.1186/s40463-020-00487-6.

## Introduction

Operating rooms (OR) are noisy. Surgical equipment, monitors, heating ventilation and air conditioning (HVAC) systems, music and team member communication all contribute to noise pollution in the OR [1–6]. OR noise pollution can negatively impact surgical technique and team communication [7, 8]. Such factors can lead to poor surgical outcomes [9, 10]. More so, acoustic trauma and noise-induced hearing loss to patients is also documented, as anesthesia can blunt natural acoustic reflexes to sudden spikes in noise [4]. Increased noise during surgery can also be deleterious for OR team members’ health. Noise-induced hearing loss (NIHL) and tinnitus are among adverse outcomes for staff with sustained exposure to loud ORs [11]. Recommendations by the World Health Organization (WHO) and Environmental Protection Agency (EPA) have established hospital and OR noise safety limits as 35 a-weighted decibels (dBA) and 45 dBA, respectively [12, 13].

Previous data has demonstrated that OR noise levels can be greater than the aforementioned safety limits, ranging between 51 and 75 dBA [12, 13]. For example, literature in Orthopedic Surgery has focused on exploring noise levels, and its detrimental effects [2, 14–16]. Otolaryngology-Head and Neck Surgery (OHNS) ORs are among some of the loudest due to the use of high-speed tools [17, 18]. Despite this, there is a dearth of literature evaluating noise in OHNS surgery. Specifically, it is unclear whether noise in OHNS surgery can negatively impact OR team communication, healthcare worker safety and patient outcomes.

As such, the purpose of this study is to quantify noise levels across all OHNS surgeries as well as OHNS specific cadaver labs. By identifying studies assessing noise OHNS ORs and cadaver labs a more unified understanding regarding what contributes to increased noise in these environments is possible. Importantly, interventions aimed at reducing noise during OHNS may be possible. With such an understanding, OR team communication, surgical performance and patient outcomes may be improved.

## Methods

### Search

This systematic review was completed in accordance with the Preferred Reporting Items for Systematic Reviews and Meta-Analyses (PRISMA) guidelines [19]. The database searches were performed by two reviewers (G.S. / A.N) and corroborated by a health sciences librarian at the University of Toronto. Databases searched included: Ovid Medline, Ovid EMBASE, Pubmed, SCOPUS and Cochrane. The search was completed from database inception (1946) to April 1, 2020. Keywords and Medical subject headings (MeSH) that were searched included: noise, sound, amplification, decibel; operating room, operating theatre, operation; communication, conversation; surgeon, scrub nurse, circulating nurse, anesthesiologist; patient morbidity; otolaryngology; head and neck surgery. Additionally, MeSH terms of 27 of the most common OHNS surgeries were included in the search (Supplementary Table 1). These surgeries were selected by the authors.

### Inclusion and exclusion criteria

Inclusion criteria consisted of studies investigating noise or sound measurements inside of OHNS ORs and/or simulations utilizing cadaveric labs. Prospective and retrospective observational studies were included. Papers published in a non-English language or a non-peer reviewed journal were excluded. Studies looking at noise in non-OHNS ORs were excluded. Abstracts, conference posters, reviews, letters to editors, editorials were also excluded.

### Data extraction and analysis

Two reviewers (G.S. / A.N.) selected articles from the search, based on the aforementioned inclusion and exclusion criteria. If there were any disagreements in article selection between the two reviewers, these were resolved by consensus. If a disagreement persisted, a third reviewer was consulted (M.L.) All title, abstract and full text screening was completed using Covidence (version 1501). Extracted data included in study demographics, noise-related data as well as any data regarding the effects of noise. Data from full text extraction were then placed into and categorized in a Google Sheets document. Means and standard deviations (SD) of noise measurements were calculated. Independent-sample t-tests were used to compare maximum (Lpeak) and mean noise measurements. *P*-values of < 0.05 were considered to be statistically significant. Descriptive results and statistical analysis were performed in Stata (version 15.1).

## Results

### Study demographics

**Fig. 1:**
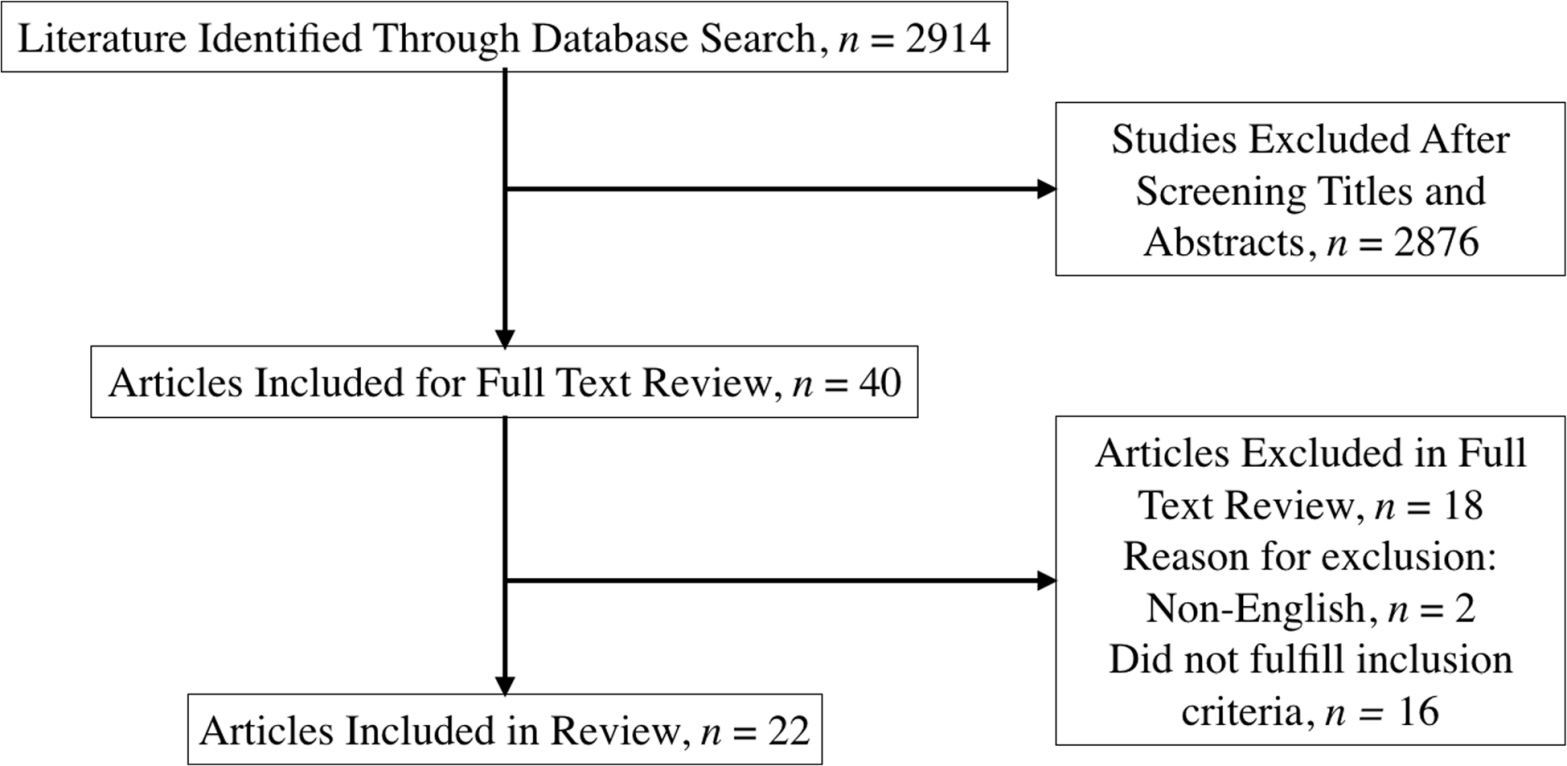
PRISMA flowchart

This search initially identified 2914 articles. Final inclusion consisted of 22 articles (Fig. 1). Ten articles quantified noise in OHNS ORs, and 11 in OHNS cadaver labs (Table 1). One study quantified noise in both settings [36]. Study demographic data are displayed in Table 1. With regards to included study homogeneity, the majority of articles analyzed noise level exposures during mastoid surgery (18/22, 82%). The other four studies included head-and-neck reconstructive procedures, neck dissections or unspecified OHNS ORs.

**Table 1:** Included article demographic data

Authors	Year of publication	Country of publication	Number of centers	Type of surgery	Type of study
Cho et al. [20]	2019	Korea	1	Cochlear implant, canal wall down mastoidectomy, intact canal wall down mastoidectomy	OR
Dalchow et al. [21]	2013	Germany	1	Temporal bone surgery	Cadaver
Hilmi et al. [22]	2011	Scotland	1	Mastoid surgery	Cadaver
Hodge and Thompson [23]	1990	Australia	1	Radical neck dissection	OR
Holmquist et al. [24]	1978	Sweden	1	Mastoid surgery	OR
Jiang et al. [25]	2007	United Kingdom	2	Mastoidectomy and tympanostomy	Cadaver
Kracht et al. [26]	2007	USA	1	N/A	OR
Kramer et al. [27]	2015	Germany	1	Osteotomy of temporal bone with intact middle ear	Cadaver
Kylen and Arlinger [28]	1976	Sweden	1	Mastoid surgery	Cadaver
Kylen et al. [29]	1977	Sweden	1	Mastoid surgery	Cadaver
Lee et al. [30]	1999	Korea	1	Mastoidectomy	OR
Man and Winerman [31]	1985	Israel	1	Mastoidectomy	OR
Michaelides et al. [32]	2001	USA	1	Temporal bone surgery	Cadaver
Parkin et al. [33]	1978	USA	1	Mastoid surgery	Cadaver
Pau et al. [34]	2007	Germany	1	Cochleostomy	Cadaver
Prasad and Reddy [18]	2003	United Kingdom	1	Mastoidectomy, endoscopic sinus surgery	OR
Stromberg et al. [35]	2010	Sweden	1	Mastoidectomy and cochleostomy	Cadaver
Tay et al. [3]	2015	United Kingdom	1	Head and neck including dentoalveolar, orthognathic, trauma, facial skin cancers, reconstructive procedure	OR
Vaisbuch et al. [36]	2018	USA	1	Temporal bone dissections in temporal bone lab and translabyrinthine resection of vestibular schwannoma	OR and Cadaver
Verhaert et al. [37]	2013	Belgium	1	Cortical mastoidectomy and posterior tympanotomy	OR
Wang et al. [38]	2017	China	1	N/A	OR
Yin et al. [39]	2011	Sweden	2	Mastoidectomy and tympanotomy	Cadaver

### Noise measurements

Tools used to quantify noise levels in OHNS ORs and OHNS cadaver labs are summarized in Table 2. Location of noise measurements are summarized in Table 3. In OHNS ORs, three studies measured noise levels from certain positions within the OR [23, 26, 38]. Five studies measured noise levels from the position of the surgeon’s ear or shoulder [18, 23, 30, 36, 37]. Three studies measured noise levels from a position close to the burr or patient’s ear [3, 24, 31] and one study did not specify the location of noise measurement [20]. In OHNS cadaver labs, five studies measured noise levels from a position on a segment of temporal bone [22, 28, 29, 32, 33] and four studies from a position within the external auditory canal (EAC) of the cadaver [27, 34, 35, 39]. In one study, the noise dosimeter was fixed to participants in the lab [36] and in another from within a silent chamber [21]. One study did not specify the location of measurement [25].

**Table 2:** Article specific noise data

Authors	Noise measurement tool	Location of measurement	Max noise level (dBA)	Background noise (dBA)	Average noise level (dBA)
Cho et al. [20]	Three B&K 2270, four LD 831c machines. A B&K Dirac System (type 7841) with a B&K 4130 microphone and a B&K 4292 omni-directional source was used for room acoustic measurement.	N/A	62. 5	N/A	49.2
Hodge and Thompson [23]	Two sound level meters (B&K 2209) and an inch remote microphone (B&K 4149 1/2′)	Centrally over operating field and level with surgeon’s ear (so that recorded sound levels were similar those heard by the surgeon)	108	13	48.3
Lee et al. [30]	Quest 2700 sound level meter	Noise produced by drilling instrument at the site of the operating ear was measured at each person’s position.	83	N/A	76.8
Man and Winerman [31]	B&K 2203 sound level meter equipped with a 1″ microphone	Sound level measurements and spectral analysis were made 0.57 cm from the burr and at the same distance from the contralateral ear during surgery	83	50	65.1
Prasad and Reddy [18]	SLM 3/IS ACOS Class I sound level meter calibrated to BS 1259	Recordings made at the level of the ear of the operating surgeon	72.4	N/A	66.7
Verhaert et al. [37]	Noise dosimeter: CR 110A doseBadge (Cirrus Research plc), Stationary sound level: NOR140 Sound Analyzer	Attached to shoulder of surgeon and surgeon assistant	109	57.7	68.1
Wang et al. [38]	Personal noise dosimeters (Aihua, Model AWA5610B)	The instrument was placed within 2 m of the anesthesia machine at a height of 1.5 m from the floor	65.8	N/A	63.3
Holmquist et al. [24]	N/A	Tape recorded drill-generated noise was delivered through an earphone fitted to the patient’s intact ear.	125.5	N/A	116.7
Kracht et al. [26]	Larson Davis System 824 sound level meter.	Instrument was placed on top of the fire extinguisher box in a corner of the theater.	115	N/A	65 dBA
Tay et al^.3^	CEMDT-8852 digital sound level meter (DigitalMeters.com, Heatmiser UK Ltd., Blackburn, UK).	Tool placed 1 m from the head of the patient.	117.4	N/A	58 dBA
Vaisbuch et al. [36]	3 M Edge EG-5 Series and 3 M NoisePro DLX personal noise dosimeters. 3 M SoundPro sound level meters used to collect general noise levels in the room	Fixed to participants (i.e. two residents, two instructors in the temporal bone lab, as well as to surgeon and scrub technician in the OR) with the microphone at ear level.	94.4	N/A	70.6
Dalchow et al. [21]	Sound level meter and special near field microphones (GH-183, McCrypt, USA)	Silent chamber in a temporal bone laboratory	76	0	65.8
Kramer et al^.34^	Hydrophone (ER 7c; Etymotic Research)	Hydrophone inserted into superior semicircular canal for sound pressure analyses	123.5	N/A	N/A
Michaelides et al. [32]	Quest 155 Sound Level Meter	1 cm from the device contact area of a prepared human cadaveric temporal bone	104.1	N/A	86.9
Yin et al. [39]	ER7C probe microphone system (Etymotic Research Inc.)	The open end of the instrument was held 0.5 cm from the bone–drill interface. During drilling of a cochleostomy open end of the silicone tube was placed so that it almost touched the round window.	130	N/A	118.8
Hilmi et al. [22]	Kamplex Audio Traveller AA220 pure tone audio-meter	Device attached to temporal bone, mastoid tip in temporal bone laboratory	105.8	N/A	104
Parkin et al. [33]	A-type 2203 sound level meter (Bruel and Kjaer) connected to a type 1613 octave filter (Bruel and Kjaer), and a type 4134 microphone and probe (Bruel and Kjaer)	Temporal bone laboratory - attached to temporal bone	107.5	N/A	80.5
Pau et al. [34]	Etymotic ER7c, Elk Grove Village, IL	Temporal bone laboratory, attached to level of round window	107.2	N/A	115.1
Kylen and Arlinger [28]	A miniature accelerometer (Briiel & Kjaer 8303, weight 3.5 g) was used as a vibration pick-up. The signal from the accelerometer was amplified (Bruel & Kjaer 2603) and fed to one channel of a tape recorder (Revox A 77, 19 cm/sec, 2-track) other channel of the tape-recorder was fed by the 1 kHz-signal from the static for	Temporal bone laboratory, attached to temporal bone	100	N/A	95 dB
Kylen et al. [29]	The signal from the accelerometer was amplified (Bruel & Kjaer 2603) and fed to a tape recorder (Revox A77). The tape recordings were analysed off-line using an octave band filter (Bruel & Kjaer 1612), connected to the amplifier (Bruel & Kjaer 2603) and level recorder (Bruel & Kjaer 2305)	Temporal bone laboratory, attached to temporal bone	96.5	N/A	N/A
Jiang et al. [25]	Sound was delivered through an ER-2 earphone (Etymotic Research, Elk Grove Village, IL) coupled to the ER1-14A ear tips (Etymotic Research), which was inserted into the ear canal.	Isolated cadaveric lab	110.4	N/A	104.2
Stromberg et al. [35]	Noise levels were recorded with an ER7C prove microphone system attached to one end of a silicone tube ER/714C	Noise recordings were obtained at the round window in a cadaver model	123.3	N/A	109.7

*N/A* Not applicable

**Table 3:** Average noise level by location

Location	Average noise level (dBA)
Patient’s/Cadaver’s temporal bone/in EAC level (*n* = 10)	95
OR personnel’s ear level (*n* = 5)	66.1
Other location in OR (*n* = 4)	74.6

**Fig. 2:**
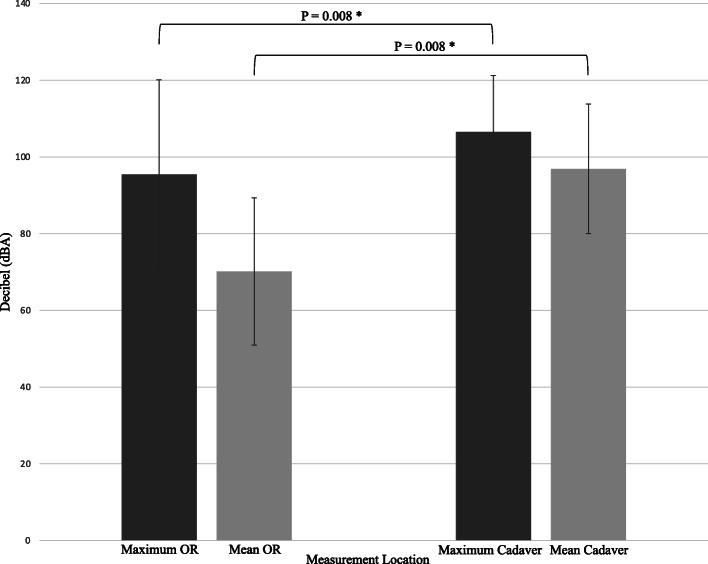
Mean and maximum noise levels by measurement location

Maximum and mean noise levels were computed for OHNS ORs and cadaver lab articles. The maximum noise level across all OHNS ORs and OHNS cadaver labs were 95.5 dBA (± SD 24.6 dBA) and 105 c-weighted decibels (dBC) (± SD 14.4 dBC), respectively (Fig. 2) (*P* = 0.2068). The range of maximum noise levels was 62.5–125.5 dBA in OHNS OR studies, and 76–130 dBA in OHNS cadaver studies. All but one article recorded mean noise levels. The mean noise levels in OHNS ORs and OHNS cadaver labs were 70.1 dBA (± SD 19.2 dBA) and 95.6 dBA (± SD 17.2 dBA), respectively (Fig. 2) (*P* = 0.0038). The mean noise level across all studies was 83.6 dBA (± SD 20 dBA) with a range of 48.3–118.9 dBA (Table 2). HVAC background noise levels were recorded in three OHNS OR studies with a mean of 42.5 dBA (± SD 20 dBA) across these articles.

### Noise exposure by procedure

The majority of the literature investigated otologic procedures, namely mastoid surgery. Four studies investigated other otolaryngology procedures that were not specified or were head and neck related surgeries including neck dissection and reconstructive surgery. Otologic procedures demonstrated significantly higher average noise levels (91.4 ± SD 19.6 dBA) in comparison to head and neck related and other otolaryngology procedures (58.6 ± SD 7.5 dBA) (*P* = 0.0046). However, there were no significant differences between mean maximum noise level of otologic surgeries (101.5 ± SD 4.7 dBC) and head and neck related and other otolaryngology procedures (101.5 ± SD 12.1 dBC) (*P* = 0.9984). Amongst the otologic procedures, noise levels were investigated in six studies from ORs during surgery [18, 20, 24, 30, 31, 37], 11 studies were performed in simulated cadaver labs [21, 22, 25, 27–29, 32–35, 39], and one study investigated both settings [36]. Average noise levels of otologic surgeries in the OR (79.3 ± SD 9.6 dBA) revealed no significant difference to cadaver simulated procedures (96.9 ± SD 5.1 dBA) (*P* = 0.096). Similarly, there was no significant difference in maximum noise levels measured in the OR (91.5 ± SD 10.7 dB) and cadaver simulation lab (106.5 ± SD 4.2 dBC) (*P* = 0.13).

## Discussion

Noise levels and exposure in operating theatres have been studied in several surgical specialties including orthopedic [1, 2, 14–16], cardiac [40], neurosurgery [26], urology [41] and general surgery ORs [42]. The literature on noise levels during OHNS surgeries have not been clearly established. This review identified, analyzed and summarized 22 articles on noise in OHNS ORs and cadaveric labs.

Recommendations by the WHO and EPA have established safe limits for noise in the OR at 45 dBA [12, 13]. Our systematic review shows that noise levels in all included studies was considerably higher than these recommendations. The Canadian Centre for Occupational Health and Safety (CCOHS) defines 87 dBA as the maximum safe noise level during an eight-hour work shift [43]. The average noise levels of included articles in this review was 83.6 dBA, nearing recommended exposure limits of 85 dBA to avoid noise-induced hearing loss (NIHL) from long term exposure [44]. The maximum noise levels in OHNS ORs was 95.5 dBA across all studies, with the highest level recorded in a study being 125.5 dBC. Maximum noise levels across all OHNS cadaver labs was 105 dBC, reaching as high as 130 dBC. Hence, while across all included studies average noise levels did not surpass recommended workplace safety levels, maximum levels were greater than proposed workplace safety levels. Average noise levels were found to be lower in OHNS ORs (70.1 dBA), compared to OHNS cadaver studies (95.6 dBA). Noise levels may have reached higher levels in simulated cadaveric studies due to the positioning of the measuring instrumentation (Table 3). For example, 9/11 of the cadaveric studies recorded noise levels from a position in close proximity to the drilling equipment, either affixed to the temporal bone or within the EAC. Whereas in OR studies, the noise measuring instruments were often placed at a central location in the OR, or at the level of the surgeon’s ear, further away from the drilling equipment. Additionally, the operator is usually 2–2.5 ft away from the drilling equipment during surgery, making the noise level quieter than what would be heard directly next to the drill. However, there was no significant difference between average noise levels in otologic procedures in OHNS OR compared to cadaveric studies, suggesting that these procedures are inherently noisy, regardless of the location in which the noise was measured. Despite differences between OR and cadaver-based studies, noise during OHNS surgery problematically reaches above the recommended WHO/EPA safety levels. As such, these noise hazards may put OHNS OR team members at risk for occupational NIHL.

Noise levels in OHNS operating theatres are considered to be among the loudest in surgical specialties [17, 18]. In Cardiac surgery, noise levels reached 90 dBA; whereas, General Surgery reached levels of 55.84 dBA [40]. Neurosurgery ORs have been reported to reach noises levels of 78.2 dBA [26]. In one study analyzing noise in orthopedic ORs, maximum sounds levels were 101.2 dBC with peak sound levels reaching as high as 134.8 dBC in total knee and total hip arthroplasty [16]. In this review, five OHNS studies showed peak noise levels ranging from 108 to 135.9 dBC. It is clear that ORs demonstrate increased levels of noise pollution, regardless of surgery type.

The literature review reveals multiple factors contributing to noise in OHNS ORs, including surgical equipment use, anesthetic monitors and background noise including laminar airflow systems and staff conversation. Suction and surgical instruments were noted to be the greatest contributors to OHNS OR-generated noise with power tools being among the noisiest instruments. Parameters further influencing noise from power tools include burr size and burr type. Burr size positively contributed to increased noise levels [20, 21, 31], meanwhile many of the studies consistently demonstrated cutting burrs producing louder noise than diamond burrs [20, 29, 39]. Among all procedures in this study, mastoid related surgery studies demonstrated significantly higher noise levels in comparison to non-mastoid related studies. This difference is likely due to the constant use of high-powered drill instruments during mastoid surgery that may not be used, or used for shorter duration, during other OHNS operations.

Noise and auditory distraction in the OR can hamper surgical performance and impair team communication. Noise has been shown to negatively impacts the surgeon’s speed of operation, time to complete surgical tasks, and the economy of the surgeon’s motion, yielding reduced accuracy and increased error rates [45]. Previous studies have reported that noise pollution during times of increased task difficulty may have an effect on surgical performance by increasing time of task completion and distance required for tool traveling during procedures [46–48]. This is most notably demonstrated by junior trainees with less surgical experience and more prone to being distracted with noise pollution.

The median expected noise-induced permanent threshold shift of 3–6 kHz at an 85-dB noise exposure level in an 8-h working day for 10 years is 4-dB, and 5-dB after 40 years. Therefore, most of the NIHL occurs in the first 10 years of noise exposure [44]. In the current review, average noise levels were 83.6 dBA when analyzed together, which closely approximates the 85-dB threshold level for risk of NIHL at 10-years. In OHNS ORs, average noise levels were 70.1 dBA, which is below that threshold. However, peak noise levels reached as high as 135.9 dBC, which may increase the risk of NIHL [32]. Additionally, certain drilling conditions, such as drilling on cortical bone with cutting burrs larger than 5 mm may pose risk to hearing [36]. Few studies have investigated the risk of occupational NIHL in OHNS ORs and with conflicting results. Prasad and Reddy concluded that powered instruments used in OHNS surgery are safe and pose no occupational hazard. While other studies have shown sound levels below international occupational noise level regulations, these authors posit that noise exposure during drilling may have negative effects on care providers [30, 36, 37]. Moreover, Fritsch et al. recorded noise levels as high as 131 dBC and concluded that instrument noise levels in average length OHNS ORs may exceed international noise regulations. Additionally, chronic exposure to noise has been linked to other chronic health pathologies such as hypertension, sleep disturbance, cardiovascular disease, anxiety, depression and others [49]. Therefore, the risk of NIHL and other chronic conditions in cumulative exposures to noise in OHNS OR may be significant and future studies should continue to elucidate this occupational hazard.

This systematic review has certain limitations. Primarily, there are few published studies on noise in OHNS ORs, and most that have been published, are focused on mastoid surgery. As such, the external validity of the results is limited due to the high noise levels generated in these procedures. For example, minor procedures performed with different equipment and limited staff may generate lower noise levels than those identified in this review. Given this shortcoming of this literature, the understanding of noise across all of the OHNS operations is still not completely understood. Although cadaveric models represent a surrogate method for measuring noise generated by various instruments and procedures, the majority of these studies measured noise from a proximity closer to what a surgeon may be exposed to in the OR. Additionally, because many of the included studies used different outcome variables, and had different potentially confounding variables, the heterogeneity of the studies made meta-analysis not possible. As no included studies were randomized-controlled trials, formal evaluation of evidence quality was not completed, however the evidence quality is likely low given the included study types. Many studies that measured noise in the OR did not quantify the impact of noise on surgical team communication and patient outcomes. Hence, while noise has been demonstrated to negatively influence OR communication in other surgical specialties, this causal relationship is still not understood in OHNS surgery., most included studies did not discuss the duration of noise exposure. As CCOHS recommends less than 87 dBA of noise consistently, for an eight-hour work shift, conclusions regarding the necessity for ear protection during OHNS ORs are limited.

Future studies on this topic should prospectively evaluate how noise in OHNS ORs contributes to miscommunication, surgical errors and patient outcomes. Moreover, certain procedures, such as those done under neuroleptic anesthesia require quiet environments. Future studies should aim to identify contributors of noise, and methods to mitigate noise exposure during these situations. Additionally, future studies should investigate noise across other OHNS ORs that commonly use drilling, such as endoscopic sinus and skull base surgery as well as OHNS minor procedures.

With the data from this study, it is important to critically evaluate how positive change can be made to reduce noise in OHNS ORs. While no studies have attempted this specifically in OHNS ORs, certain noise-reducing interventions in healthcare settings have been described that may be transferrable to OHNS. For example, Engelmann et al. demonstrated behavior modification and a noise reduction intervention program reduces pediatric OR sound intensity by 50%. Additionally, these interventions significantly lowered postoperative complications [50]. Similarly, Hogan and Harvey used personnel-specific education for OR staff members and significantly reduced noise in the OR [51]. Cabrera and Lee suggest that hospital systems implement a Department of Sound to continuously asses and evaluate noise in the hospital and search for institution-specific ways to remedy such noise [52]. From an environment engineering perspective, West et al. successfully employed sound absorptive panels into the OR to reduce noise whilst maintaining speech intelligibility and operating sterility [53]. In non-surgical noisy environments such as aviation and the military, wireless in-ear devices are used to improve communication. The concept of a similar wireless in-ear modality for the OR has been recently described by Levin and Lee in hopes to alleviate noise contributing to miscommunication [54]. Hence, available strategies to reduce noise in the OR do exist. With the data from this review, it is clear that OHNS ORs could greatly benefit from noise reduction through both the aforementioned strategies as well as continued future OR innovation.

## Conclusion

This review demonstrated that OHNS ORs are exposed to high noise levels. Such noise may have detrimental consequences to patient outcomes by impairing communication and performance amongst OR team members. Furthermore, operating theatre staff may be at risk of NIHL with repeated exposures to high noise levels. This review demonstrates that noise within the OHNS OR exceeds current safety levels set by the WHO and EPA. Most of the included studies involved mastoid surgery, which involves the use of loud drilling instruments. Further research should aim to understand how noise in OHNS ORs affect team communication and surgical outcomes. Importantly, strategies to mitigate noise pollution in OHNS ORs should be explored and implemented.

## Supplementary Information

**Additional file 1: Table S1.** MeSH Terms used in Database Searches.

## Data Availability

All data generated or analyzed during this study are included in this published article.

## Abbreviations

OR: Operating rooms
OHNS: Otolaryngology – head and neck surgery
NIHL: Noise-induced hearing loss
WHO: World Health Organization
EPA: Environmental Protection Agency
EAC: External auditory canal
dBA: A-weighted decibels
PRISMA: Preferred Reporting Items for Systematic Reviews and Meta-Analyses
MeSH: Medical subject headings
SD: Standard deviations
dBC: C-weighted decibels
CCOHS: Canadian Centre for Occupational Health and Safety

## References

[1] Siverdeen Z, Ali A, Lakdawala AS, McKay C (2008). Exposure to noise in orthopaedic theatres - do we need protection?. Int J Clin Pract.

[2] Sydney SE, Lepp AJ, Whitehouse SL, Crawford RW (2007). Noise exposure due to orthopedic saws in simulated total knee arthroplasty surgery. J Arthroplast.

[3] Tay BD, Prabhu IS, Cousin CHS, Cousin GCS (2016). Occupational exposure to noise in maxillofacial operating theatres: an initial prospective study. Br J Oral Maxillofac Surg.

[4] Katz JD (2014). Noise in the operating room. Anesthesiology..

[5] Murthy VSSN, Malhotra SK, Bala I, Raghunathan M (1995). Detrimental effects of noise on anaesthetists. Can J Anaesth.

[6] Way TJ, Long A, Weihing J (2013). Effect of noise on auditory processing in the operating room. J Am Coll Surg.

[7] Hasfeldt D, Laerkner E, Birkelund R (2010). Noise in the operating room-what do we know? A review of the literature. J Perianesthesia Nurs.

[8] Kurmann A, Peter M, Tschan F, Mühlemann K, Candinas D, Beldi G (2011). Adverse effect of noise in the operating theatre on surgical-site infection. Br J Surg.

[9] Lear R, Riga C, Godfrey AD (2016). Multicentre observational study of surgical system failures in aortic procedures and their effect on patient outcomes. Br J Surg.

[10] Greenberg CC, Regenbogen SE, Studdert DM (2007). Patterns of communication breakdowns resulting in injury to surgical patients. J Am Coll Surg.

[11] Willett KM (1991). Noise-induced hearing loss in orthopaedic staff. J Bone Jt Surg - Ser B.

[12] Berglund B, Lindvall T, Schwela DH. GUIDELINES FOR COMMUNITY NOISE: World Heal Organ; 1999.

[13] Agency USEP (EPA) (1974). Information on levels of environmental noise requisite to protect public health and welfare with an adequate margin of safety.

[14] Nott MR, West PDB (2003). Orthopaedic theatre noise: a potential hazard to patients. Anaesthesia..

[15] Fitzgerald G, O’donnell B (2012). In somno securitas anaesthetistss noise exposure in orthopaedic operating theatres. Ir Med J.

[16] Love H (2003). Noise exposure in the orthopaedic operating theatre: a significant health hazard. ANZ J Surg.

[17] Fritsch MH, Chacko CE, Patterson EB (2010). Operating room sound level hazards for patients and physicians. Otol Neurotol.

[18] Prasad KRS, Reddy KTV (2003). Live recordings of sound levels during the use of powered instruments in ENT surgery. J Laryngol Otol.

[19] Moher D, Shamseer L, Clarke M (2016). Preferred reporting items for systematic review and meta-analysis protocols (PRISMA-P) 2015 statement. Rev Esp Nutr Humana y Diet.

[20] Cho WH, Jeong CH, Chang JH (2019). Noise and room acoustic conditions in a tertiary referral hospital, Seoul National University Hospital. J Audiol Otol.

[21] Dalchow CV, Hagemeier KC, Muenscher A, Knecht R, Kameier F (2013). Investigation of noise levels generated by otologic drills. Eur Arch Oto-Rhino-Laryngology.

[22] Hilmi OJ, Mckee RH, Abel EW, Spielmann PM, Hussain SSM (2012). Do high-speed drills generate high-frequency noise in mastoid surgery?. Otol Neurotol.

[23] Hodge B, Thompson JF (1990). Noise pollution in the operating theatre. Lancet..

[24] Holmquist J, Oleander R, Hallén O (1979). Peroperative drill-generated noise levels in ear surgery. Acta Otolaryngol.

[25] Jiang D, Bibas A, Santuli C, Donnelly N, Jeronimidis G, O’Connor AF (2007). Equivalent noise level generated by drilling onto the ossicular chain as measured by laser doppler vibrometry: a temporal bone study. Laryngoscope..

[26] Kracht JM, Busch-Vishniac IJ, West JE (2007). Noise in the operating rooms of Johns Hopkins Hospital. J Acoust Soc Am.

[27] Kramer FJ, Bornitz M, Zahnert T, Schliephake H (2015). Can piezoelectric ultrasound osteotomies result in serious noise trauma?. Int J Oral Maxillofac Surg.

[28] Kylén P, Arlinger S (1976). Drill-generated noise levels in ear surgery. Acta Otolaryngol.

[29] Kylén P, Stjernvall JE, Arlinger S (1977). Variables affecting the drill-generated noise levels in ear surgery. Acta Otolaryngol.

[30] Lee HK, Lee EH, Choi JY, Choi HS, Kim HN (1999). Noise level of drilling instruments during mastoidectomy. Yonsei Med J.

[31] Man A, Winerman I (1985). Does drill noise during mastoid surgery affect the contralateral ear?. Am J Otolaryngol.

[32] Michaelides EM, Kartush JM (2001). Implications of sound levels generated by otologic devices. Otolaryngol Head Neck Surg.

[33] Parkin JL, Wood GS, Wood RD, Mccandless GA (1980). Drill- and suction-generated noise in mastoid surgery. Arch Otolaryngol.

[34] Pau HW, Just T, Bornitz M, Lasurashvilli N, Zahnert T (2007). Noise exposure of the inner ear during drilling a cochleostomy for cochlear implantation. Laryngoscope..

[35] Strömberg AK, Yin X, Olofsson Å, Duan M (2010). Evaluation of the usefulness of a silicone tube connected to a microphone in monitoring noise levels induced by drilling during mastoidectomy and cochleostomy. Acta Otolaryngol.

[36] Vaisbuch ÃY, Alyono ÃJC, Kandathil ÃC, Wu SH, Fitzgerald ÃMB, Jackler ÃRK. Occupational noise exposure and risk for noise-induced hearing loss due to temporal bone drilling. 2018;5:693–9. 10.1097/MAO.0000000000001851. 10.1097/MAO.0000000000001851 29889779

[37] Verhaert N, Moyaert N, Godderis L, Debruyne F, Desloovere C, Luts H (2013). Noise exposure of care providers during otosurgical procedures. B-ENT..

[38] Wang X, Zeng L, Li G (2017). A cross-sectional study in a tertiary care hospital in China: noise or silence in the operating room. BMJ Open.

[39] Yin X, Strömberg AK, Duan M (2011). Evaluation of the noise generated by otological electrical drills and suction during cadaver surgery. Acta Otolaryngol.

[40] Ginsberg SH, Pantin E, Kraidin J, Solina A, Panjwani S, Yang G (2013). Noise levels in modern operating rooms during surgery. J Cardiothorac Vasc Anesth.

[41] Ayoub CM, Rizk LB, Yaacoub CI, Gaal D, Kain ZN (2005). Music and ambient operating room noise in patients undergoing spinal anesthesia. Anesth Analg.

[42] Keller S, Tschan F, Semmer NK (2018). Noise in the operating room distracts members of the surgical team. An observational study. World J Surg.

[43] Government of Canada CC for OH and S. Noise - Occupational Exposure Limits in Canada : OSH Answers. 2020. [cited 2020 Jun 8]. Available from: https://www.ccohs.ca/.

[44] Lie A, Skogstad M, Johannessen HA (2016). Occupational noise exposure and hearing: a systematic review. Int Arch Occup Environ Health.

[45] Mentis HM, Chellali A, Manser K, Cao CGL, Schwaitzberg SD (2016). A systematic review of the effect of distraction on surgeon performance: directions for operating room policy and surgical training. Surg Endosc.

[46] Suh IH, Chien JH, Mukherjee M, Park SH, Oleynikov D, Siu KC (2010). The negative effect of distraction on performance of robot-assisted surgical skills in medical students and residents. Int J Med Robot Comput Assist Surg.

[47] Siu KC, Suh IH, Mukherjee M, Oleynikov D, Stergiou N (2010). The impact of environmental noise on robot-assisted laparoscopic surgical performance. Surgery..

[48] Feuerbacher RL, Funk KH, Spight DH, Diggs BS, Hunter JG (2012). Realistic distractions and interruptions that impair simulated surgical performance by novice surgeons. Arch Surg.

[49] Stansfeld SA, Matheson MP (2003). Noise pollution: non-auditory effects on health. Br Med Bull.

[50] Engelmann CR, Neis JP, Kirschbaum C, Grote G, Ure BM (2014). A noise-reduction program in a pediatric operation theatre is associated with surgeon’s benefits and a reduced rate of complications: a prospective controlled clinical trial. Ann Surg.

[51] Hogan LJ, Harvey RL (2015). Creating a culture of safety by reducing noise levels in the OR. AORN J.

[52] Cabrera IN, Lee MHM (2000). Reducing noise pollution in the hospital setting by establishing a department of sound: a survey of recent research on the effects of noise and music in health care. Prev Med.

[53] West J, Busch-Vishniac I, King J, Levit N (2008). Noise reduction in an operating room: a case study. J Acoust Soc Am.

[54] Levin M, Lee Y (2019). A novel wireless in-ear device for surgical care: an innovative idea to improve operating room miscommunication. Surg Innov.

