# Supplementary material for: Noise in Otolaryngology – Head and Neck Surgery operating rooms: a systematic review
Source: J Otolaryngol Head Neck Surg. 2021 Feb 11;50:8. doi: 10.1186/s40463-020-00487-6 (PMC7879658; doi:10.1186/s40463-020-00487-6)
Supplement: Supplementary file 1 — Additional file 1: Table S1. MeSH Terms used in Database Searches. [file 40463_2020_487_MOESM1_ESM.docx]

**Supplementary Table 1.** **MeSH Terms used in Database Searches**

| Concept | MeSH Term |
| --- | --- |
| Noise related terms | Noise, sound, amplification, decibel |
| Operating theatre related terms | Operating room, operating theatre, operation; communication, conversation; surgeon, scrub nurse, circulating nurse, anesthesiologist; patient morbidity; |
| Most common OHNS surgeries | Head and neck surgery; thyroidectomy, parathyroidectomy, parotidectomy, submandibulectomy, mandibulectomy, neck dissection, tonsillectomy, adenoidectomy, otologic surgery, myringotomy, pharyngectomy, glossectomy, laryngectomy, laryngoplasty, sinus surgery, functional endoscopic sinus surgery, skull base sinus surgery, rhinoplasty, septoplasty, otosurgical procedures, stapedectomy, mastoidectomy, myringoplasty, tympanoplasty, craniofacial surgery, cochlear implantation |
